# Getting the Most From Your Extreme Wind Data: A Step by Step Guide

**DOI:** 10.6028/jres.099.038

**Published:** 1994

**Authors:** David Walshaw

**Affiliations:** University of Newcastle upon Tyne, Newcastle upon Tyne, U.K.

**Keywords:** extreme value theory, generalized pareto distribution, peaks over threshold, return levels, statistics of extremes, wind speed

## Abstract

Models for extremes of environmental processes have been studied extensively in recent years. The particular problems arising when attempting to estimate return levels from sequences of measurements on the appropriate variables have been considered in some detail. In particular, the aspects of seasonal variation and short-range dependence have received a great deal of attention. In this paper we present a case study based on 10 years of hourly wind speed measurements collected at a U.K. site, elucidating the most successful procedure emerging from an extensive study of this data. The basic model (in which an extreme value distribution is fitted to cluster peak excesses over a high threshold) is standard. However the emphasis is on a number of practical problems which will arise when such models are fitted to wind speeds, but which have received little consideration. These include: model selection and assessment of model adequacy when the threshold, and some or all of the parameters, are allowed to vary seasonally; the choice of the best combination of threshold and cluster identification procedure; and the choice of a measure of precision for return level estimates. The aim is to suggest an algorithm which can be generally applied to the problem of gust return level estimation at individual sites.

## 1. Introduction

Threshold models for exceedances have been widely adopted in recent years in the study of extremes of environmental processes. The main advantage of such models over the so-called “classical” extreme value models (in which a limiting distribution is fitted to the largest order statistics selected form fixed time intervals) is their greater flexibility in the manner in which events are identified as “extreme.” This generally leads to a larger number of extreme events being available for analysis, and this in turn to more precise estimates for return levels and return periods.

The price paid for the increased efficiency of data exploitation and consequent improvements in estimation precision is, as one would expect, a greater complexity of model. Seasonal variation and short-range correlation, almost always present in environmental time series, can no longer be ignored in the manner of a traditional “annual maxima” analysis (or “Gumbel analysis”). Instead they must be given careful consideration. Models which take account of both of these features have received considerable attention in the literature (e.g., Refs. [2,3,7,8] and the associated discussion).

In this paper we consider a complete study of a sequence of wind speed measurements recorded at a single U.K. site. We address some of the practical complexities that arise when adopting a threshold-based approach to extremes of environmental time series. In particular, the related issues of

1. choosing a threshold large enough for the distribution of excesses to approximate to a limiting form,
2. allowing the threshold and some or all of the parameters to vary seasonally,
3. employing a threshold-based declustering method for identification of storm peaks,

give rise to a situation which requires some careful consideration in terms of the practical application of existing models.

The theoretical arguments supporting the use of threshold models in the manner considered in this paper, already validated in previous studies (e.g., Ref. [8]), suggest that the techniques employed should be applicable at any site at which the natural mechanism underlying the generation of extreme winds is not capable of taking on several distinct forms (e.g., hurricanes and conventional storms). Thus the approach considered here could be viewed as a possible algorithm for the estimation of the extreme wind potential at any site in a temperate climate.

## 2. Background to the Study

### 2.1 Wind: The Variable

The behaviour of wind velocity as a continuous variable demonstrates certain characteristics which distinguish it from other environmental variables. In common with other such variables, clear seasonal patterns and short-range dependence are strong features of the wind climate at most locations. However, in comparison with these others, wind velocity is fairly well-behaved in a number of ways. Unlike sea-level (Ref. [9]), wind speed does not naturally break down into distinct components, and unlike rainfall, the wind does not arrive in clearly identifiable episodes. In comparison with many environmental phenomena, wind velocity is not subject to *very* violent departures from the norm. Although a wind velocity of 200 mph may sound rather severe, from a statistical point of view such departures from mean levels are small compared with those occasionally demonstrated by rainfall levels over short periods, flow rates in rivers, and concentrations of certain pollutants. The relative stability of wind velocity is more akin to sea-level behaviour, but wind speed differs from sea-level in being one of the most rapidly varying of all environmental variables. Conditional on the underlying “level” of the wind (characterized by storms and periods of calm), many distinct gusts can be observed in periods as short as several minutes. In a sense therefore, while being rather stable, the wind can provide us with a great deal of information in a relatively short time. This strengthens arguments supporting limiting asymptotic distributions for the most extreme gusts, and potentially allows us to make inferences about long-period return levels from comparatively short runs of measurements.

### 2.2 Extreme Value Models: Exploiting the Variable

We consider the problem of estimating gust return levels for specified periods of the order of 50 or more years, when data available consist of recorded maximum gusts taken over short intervals (say 1 hour or 1 day), and are collected over a time period which may be short in comparison to the return periods (perhaps less than 10 years). In such situations, a classical approach based on annual maxima is unworkable, due to sparsity of data. Methods which make use of several order statistics from each year (for example the “*r* largest” approach advocated by Tawn [9] in analysing extreme sea-levels) can produce viable estimates of 50 year gust return levels from as little as 10 years of data [10]. However, such methods must take account of serial correlation, and are vulnerable to the effects of seasonal variation. Seasonal effects could be incorporated into the models, but given the additional complexity this would entail, it is thought preferable to convert to a threshold-based approach. The main advantage over the use of order statistics from fixed time intervals is the additional flexibility in the choice of extreme events for analysis. This arises from allowing the number of such events which occur over a fixed period to vary according to the behaviour of the wind during that time. Serial correlation can be dealt with by identifying clusters of observations above a threshold, which are deemed to be correlated, and discarding all but the largest observation within each cluster. The aim here is to filter out a set of independent “cluster peak excesses” for further analysis (Ref. [7]). Seasonal variability in the behaviour of extremes can be incorporated by allowing the threshold (above which events are deemed to be extreme), and the distribution of excesses over this threshold, to vary through the year. However the justification for such a model is not immediate and is worth considering in a little more depth.

It is usual in strongly seasonal climates for the occurrence of truly extreme wind speeds to be confined to a certain part of the yearly cycle. In the U.K. for example, it is very unusual for wind damage to occur outside the period October through March. However a model for extreme values which *takes account* of this seasonality will select as extreme events all gusts which are large *given the time of year*. If the probability of important levels being exceeded during certain seasons is negligible, then there is only a point to modelling the extremes observed during these periods if we believe that they can supply additional information about what may happen in the seasons in which genuinely large events *can* occur. For this to be the case, we must assume that there is some homogeneity in the extremal behaviour across the different seasons—that in some sense it is fundamentally the same mechanism which is responsible for the generation of large gusts throughout the year, and it is just some of the associated parameters of this mechanism which change. Fortunately, there are often good reasons for making this assumption. In temperate climates, it is essentially the same alternating passage of anticyclones and depressions which leads to all the storms which occur throughout the year. It is merely the severity of these systems which is seasonally variable. Hence it seems reasonable to assume that the manner in which large events cluster together will be broadly homogeneous throughout the year.

A further, more tentative contention is that the patterns of turbulence caused by the local terrain around a site also remain essentially unchanged throughout the annual cycle. Since it is this turbulence that is the cause of gusting, i.e., very short term fluctuations away from the mean wind speed, and since the systems generating sequences of high or low mean speeds appear to differ from season to season only in their severity, we suggest that the *shape* of the upper tail in the distribution of gusts could well be homogeneous throughout the year (i.e., the distribution of extremes varies seasonally only in terms of location and scale). In terms of fitting extreme value distributions to large gusts, this would be reflected by the shape parameter (denoted here by *k*) being held constant across all seasons.

Of course homogeneity conditions on both clustering behaviour of large gusts, and the shape of the upper tail in their distribution, must be verified from the data. However, previous studies suggest that such assumptions are often validated, and can then provide an important route to a more efficient exploitation of data. This will be demonstrated in the case study which follows. Working with hourly maximum gusts collected at Sheffield University for the U.K. Meteorological Office over a 10 year period 1975–1984, we identify four steps to the estimation of return levels. Implementing this algorithm, we obtain useful return level estimates for 10, 50, and 1000 years. The level of precision attached to these estimates is greater than any achieved via a whole range of conventional analyses applied to the same data, as well as some more novel models (see Ref. [10]).

## 3. Step 1 — Generating a Stationary Series

### 3.1 Dealing with Seasonal Variation

Davison and Smith [3] identify two basic approaches for handling seasonal data:

1. the removal of known seasonal components to create a stationary (prewhitened) series;
2. a separate seasons approach, in which a different model is fitted within each of a finite number of seasons.

For wind-speed data there are no clearly defined seasonal components. Also, as Davison and Smith [3] point out, it is important that the seasonal effects identified are those which affect the upper tails, rather than the central portion of the data. We therefore advocate the separate seasons approach (with a different extreme value model being fitted to large gusts from each season) as the more natural choice. However, as stated in Sec. 2, we hope to be able to exploit homogeneities across seasons in the mechanisms underlying generation of extreme gusts. This may involve application of a uniform procedure for identification of clusters of large observations, and/or the fitting of a constant shape parameter across all seasons. Now the assessment of goodness-of-fit of extreme value models generally entails graphical rather than formal methods, due to the intractability of the latter, and the ease of application and interpretation of the former. In particular, the mean excess plot [mean residual life (MRL), or conditional mean excess (CME)] is advocated for the limiting Generalized Pareto Distribution (GPD) fitted to threshold excesses (see Lechner, Leigh, and Simiu [5,6] for arguments in justification). In order to check our homogeneity assumptions we must be able to assess the adequacy of the model to all the seasons *simultaneously*. For this purpose we suggest the generation of a prewhitened series for the preliminary stages of the analysis *only*, namely the choice of an appropriate seasonally varying threshold, an accompanying method of identifying clusters of observations above this threshold, and the initial assessment of model adequacies.

In this paper, we take our seasonal unit to be 1 month. Experience suggests that by dividing the year into 12 equal-length seasons, we strike a good balance between the two conflicting requirements of a) reflecting reasonably accurately the continuous nature of seasonal changes in climate, and b) retaining a substantial amount of data for analysis within each season. The models we will consider thus consist of a separate GPD fitted to cluster peak excesses within each month, the threshold also being allowed to vary on a monthly basis. We will assume a homogeneous clustering mechanism throughout the year, but retain the option of allowing the shape parameter *k* to vary from month to month, or constraining it to take a single value across all months. (In other situations where a different length of season is considered appropriate, the arguments laid out below would apply unchanged.)

Under such a separate months model, an appropriate set of prewhitening operations would be provided by separate transformations *t_m_* for each month *m* (applied to all the observations in month *m*). In order to know the precise transformations required, we would need to know the parameters in the GPDs fitted to cluster peak excesses within each month. Since we have not yet established how to obtain the cluster peak excesses (CPEs), we cannot know these values. However it is possible to make an educated guess at an appropriate set of monthly transformations, as shown in the following sections.

#### 3.1.1 Homogeneous Shape Parameter *k*

We consider first the situation in which the GPD shape parameter *k* is assumed constant over all months. It is then easy to show that a set of *linear* transformations *t_m_*(*x*) *= a_m_x +b_m_*; *a* >0, *m* = 1, …, 12 can be chosen to render the distribution of CPEs over a *single* threshold homogeneous GPD across all months (see Ref. [10]).

In order to form estimates for the required transformations, we bear in mind that it is the upper tails of the monthly distributions of all recorded maximum gusts (in our case hourly) which will yield the CPEs. Since the clustering mechanism is assumed homogeneous across all months, we suggest that a good approximation to the appropriate transformations will be obtained by making the upper tails of the empirical monthly distributions of all recorded maximum gusts coincide with each other in some sense. Since the required transformations are linear, this can be achieved by transforming two high quantiles (e.g., 0.95 and 0.99) from each month to two distinct arbitrarily specified points, say the corresponding theoretical quantiles of the unit exponential distribution. Explicitly, we would transform empirical monthly quantiles *z*_1_*_m_* and *z*_2_*_m_* to the corresponding exponential quantiles *q*_1_ and *q*_2_ by solving the simultaneous equations:

$$\begin{array}{l} a_{m}z_{1m}+b_{m}=q_{1}\\ a_{m}z_{2m}+b_{m}=q_{2} \end{array}$$ (1)

for *a_m_* > 0 and *b_m_*, and for each *m* = 1, …, 12. The precise choice of quantiles is not critical, and is somewhat arbitrary. It is determined by the necessity of moving as far as possible into the upper tails, while still retaining a substantial amount of data between the two quantiles, and above the largest of them (in order to keep sampling error to a minimum).

#### 3.1.2 Variable Shape Parameter *k*

If the shape parameter *k* is allowed to vary from month to month, the required monthly transformations are no longer linear. However, the arguments leading to approximately the correct transformations being obtained (by causing the upper tails of the empirical monthly distributions of all monthly gusts mutually to coincide) still hold. This time transformations which will lead to monthly cluster peak exceedances being homogeneous GPD over a single threshold are of the form *t*_m_(*x*) = *a_m_*log(*x* − *c_m_*) + *b_m_*; *a_m_* > 0 (easily obtained by considering the transformation which maps one GPD c.d.f. onto another). Estimates can be obtained by transforming *three* high quantiles (e.g., 0.90, 0.95, and 0.99) from each month to distinct arbitrary points. For example the empirical quantiles *z*_1_*_m_*, *z*_2_*_m_*, and *z*_3_*_m_* from each month *m* could be transformed to the corresponding theoretical quantiles *q*_1_, *q*_2_, and *q*_3_ of the unit exponential distribution by (numerically) solving the simultaneous equations:

$$\begin{array}{l} a_{m}\log\left(z_{1,m}-c_{m}\right)+b_{m}=q_{1}\\ a_{m}\log\left(z_{2,m}-c_{m}\right)+b_{m}=q_{2}\\ a_{m}\log\left(z_{3,m}-c_{m}\right)+b_{m}=q_{3} \end{array}$$ (2)

for *a_m_* > 0, *b_m_*, and *c_m_*, and for each *m* = 1, …, 12.

### 3.2 Implementation for the Sheffield Data

For each month, 10 years of hourly maximum gusts constitute approximately 7300 observations. Hence there are about 365 points lying above the 0.95 quantile; 73 above the 0.99 quantile. The sampling error in estimating these quantiles’ theoretical values via the empirical equivalents is therefore reasonably small. We initially make the assumption of a homogeneous shape parameter. As we shall see in Sec. 4, this appears to be well-founded. For each month, then, linear transformations which map the two empirical quantiles to their theoretical unit exponential counterparts (2.996 and 4.605), are applied to all hourly maxima. The resulting prewhitened sequence occupies the range [−4.559, 8.596].

## 4. Step 2—Threshold Selection

### 4.1 Methodology

Having created an approximately stationary (in the upper tail at least) sequence of hourly maximum gusts, we are in a position to experiment with various choices of threshold and cluster identification procedure.

We propose a constant threshold for the prewhitened series, based on the assumption that the region of the data to be treated as extreme will constitute the same upper quantile for all seasons. Applying the inverses of the prewhitening transformations to this threshold in monthly segments will then provide the seasonally varying threshold for use in the final model.

Exceedances of the threshold will occur in clusters (storms) from which we wish to choose only the peak excesses for modelling. We need to be able to identify these clusters, bearing in mind that some of the observations within a storm may lie below the threshold. Of several possible methods, we opt for a fixed termination time approach, whereby a storm is deemed to have ended when a certain required number of consecutive observations below the threshold are observed. The advantage of this method over some others is that it allows both the duration of storms, and the duration of intervals between them to vary according to the data, reflecting the inherent natural variability of these quantities.

The threshold and the termination interval may be regarded as the two parameters for estimation in this section of the analysis. Formal estimation procedures such as maximum likelihood are inappropriate here: distributional assumptions on CPEs only hold if the threshold is chosen high enough, and we do not wish to impose a specific model structure on the underlying process which generates storms and periods of calm. However, graphical procedures are highly effective in this capacity. In particular the mean excess plot (the mean residual life plot: see Ref. [4]; or conditional mean excess plot: e.g., Ref. [5,6]) performs well. This is produced by simply plotting the mean excess of all model data above threshold *u* against *u* for a range of such thresholds. Linearity in the plot corresponds to a good fit of the GPD to excesses of the model data over any threshold above which the linearity holds. In our case the model data will be the selected cluster peak exceedance magnitudes.

Note that the threshold and the termination interval must be chosen in combination, because these two parameters interact in the manner in which they determine the set of cluster peak exceedances actually selected. Basically, provided both are large enough, the set of corresponding CPEs should be iid GPD, because

1. the GPD exhibits a threshold stability property, whereby a good fit above a certain threshold implies a good fit above all higher thresholds, with merely a change in scale parameter, and
2. if the termination interval is long enough for the CPEs to be approximately independent, then this will still hold for increased intervals.

However, subject to this constraint, we wish to make both quantities as *small* as possible, in order to maximize the number of valid CPEs selected for analysis.

In principle, it would be possible to produce a large number of mean excess plots to examine the model adequacy under a whole variety of combinations of threshold and termination interval. In practice however, this would prove a very cumbersome route to making an appropriate choice. Instead we propose a simple modification to the mean excess plot which leads to considerable streamlining of the selection procedure. For a given termination interval z*, we propose that the mean excess above threshold *u* is plotted against *u*, *with the identification of cluster peak exceedances being carried out separately for each threshold u*. We will call this device a *reclustered excess plot*. The idea here is that linearity in such a plot above a certain threshold *ū* suggests both a good fit of the GPD to CPEs over *ū* selected using termination interval z*, *and* a robustness of the mean CPE to the threshold at which *declustering* is carried out. Note that if such a robustness were *not* present, it would cast considerable doubt on the validity of the declustering procedure.

By producing individual reclustered excess plots for a range of values of z* (each one requires surprisingly little computation time), we should be able to identify the smallest such value for which the independence criterion for the CPEs is met to a good approximation. This will be the smallest value yielding a plot which straightens out above a certain level *ū*. This value of *ū* is then chosen as the best threshold for the corresponding value of z*, giving the optimal pairing (*ū*, z*).

Note that having selected the pair (*ū*, z*), it is strongly recommended that a conventional mean excess plot is obtained for the CPEs so obtained, the plotting range being *u ≥ ū*. This is to verify the validity of the choice, and in particular to check that approximate linearity in the reclustered excess plot is not caused by lack of fit of the GPD and non-robustness to the declustering threshold having opposing effects, and thereby cancelling one-another out.

For a more in-depth discussion of reclustered excess plots and their validity, see Ref. [10].

We suggest that we first work with a prewhitened series obtained under the assumption of a homogeneous shape parameter *k*, since this will provide a very useful improvement in return level estimation precision if it proves to be justified. Only if the reclustered and mean excess plots suggest a poor fit for all trial values of z* do we recommend relaxing this assumption and working with a prewhitened series created using non-linear transformations.

Note that the effect of a moderate failure in the assumption of homogeneous *clustering* behaviour is not liable to be serious. While this implies that z* should be allowed to vary seasonally, the above procedure will tend to lead to the selection of the smallest z* value large enough to work for *all* seasons: any smaller value of z* will fail in some parts of the annual cycle, and this should show up as a lack of fit of the overall GPD model to CPEs from the prewhitened series.

### 4.2 Implementation for the Sheffield Data

**Fig. 1:**
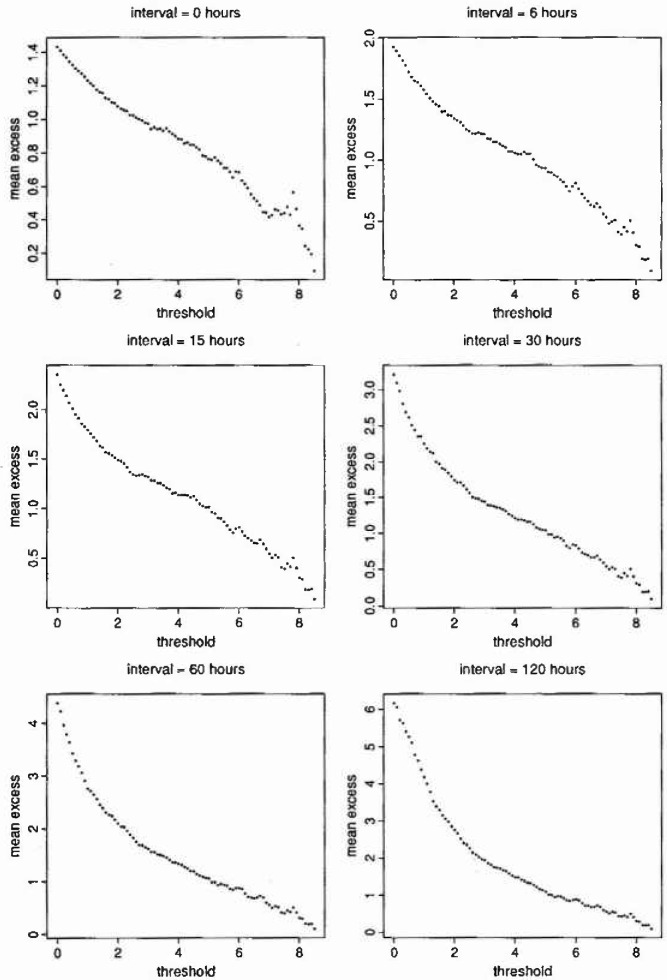
Reclustered excess plots.

**Fig. 2:**
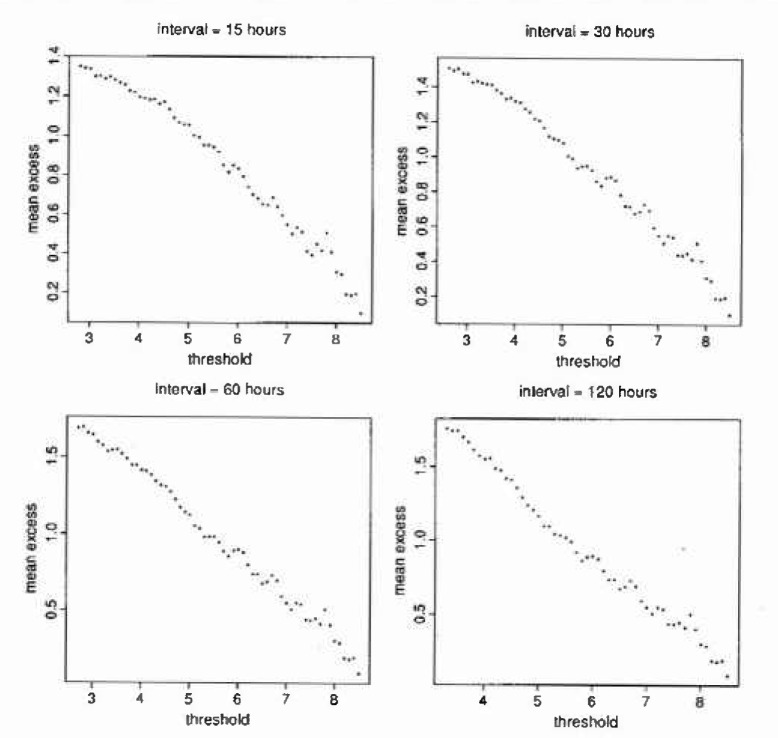
Mean excess plots.

Figure 1 shows reclustered excess plots produced for termination intervals z* = 0 (all excesses), 6 h, 15 h, 30 h, 60 h, and 120 h. Here we are using the prewhitened series obtained at the end of Sec. 3, based on the homogeneous *k* assumption. The plots appear to straighten for z* = 30 h (debatable), 60 h, and 120 h, but not for the smaller termination intervals. Conventional mean excess plots (Fig. 2) produced for z* = 15 h, 30 h, 60 h, and 120 h using the corresponding linearity thresholds *ū* = 2.8, 2.6, 2.7, and 3.3 (for z* = 15 h we use the inflection point) broadly support the findings, and we conclude that z* = 15 h is too small; z* = 30 h is borderline; and z* = 60 h or z* = 120 h is large enough.

The fact that the fit of a single GPD to this prewhitened series appears good supports the homogeneity assumption on *k*, and we do not need to abandon this in favour of a model which allows *k* to vary.

We select the pairs (*ū* = 2.6, z* = 30) and (*ū* = 2.7, z* = 60) as our choices for the next stage of modelling. We retain *two* combinations because of the doubt over the adequacy of the termination interval z* = 30 h, and in order to check on the robustness of final results to the precise choice of CPEs. The 10 years of hourly maximum gusts yield respectively 525 and 352 CPEs under the two pairings. The thresholds 2.6 and 2.7 lie at the 0.923 and 0.935 quantiles in the empirical distribution of transformed hourly maxima.

## 5. Step 3—Model Verification

### 5.1 Likelihood Ratio Tests

From any given choice of threshold and termination interval, and the corresponding monthly sets of cluster peak exceedances, we are able to move directly to a separate seasons model for the raw (untransformed) cluster peak exceedances. Under the appropriate model, the excesses of these in month *m* over a segmented monthly varying threshold (obtained by applying the inverses of the prewhitening transformations to the threshold *ū* identified in Sec. 4) are independent GPD(*σ_m_*,*k_m_*), with distribution functions

$$G_{m}\left(y;\sigma_{m},k_{m}\right)={\left(1-k_{m}y/\sigma_{m}\right)}^{1/k_{m}};$$ (3)

scale parameters *σ_m_* >0; shape parameters *k_m_* arbitrary; and *G_m_* defined on 0 < *y* < ∞ if *k_m_ ≤* 0, and 0 < y < *σ_m_*/*k_m_* if *k_m_* > 0. The case *k_m_* = 0 is interpreted as the limit *k_m_*→0, and is the exponential distribution with mean *σ_m_*. The parameters *σ_m_* and *k_m_* can be estimated via numerical maximum likelihood estimation. (N.B. at this stage of the modelling, the values *u_m_* are treated as fixed constants. Starting values for *σ_m_* and *k_m_* can be provided from the graphical estimates for scale and shape parameters for the prewhitened CPEs obtained using the fact that the fitted line on the mean excess plot should have slope −*k/*(1 *+ k*) and intercept *σ*/(1 *+ k*); see Ref. [3]. Applying the inverses of the prewhitening transformations to the GPD(*σ*,*k*) will give good preliminary estimates for *σ_m_* and *k_m_*). It is then possible to verify the choice of homogeneous or variable shape parameter *k* via a likelihood ratio test—twice the decrease in fitted log-likelihood when *k* is constrained to be homogeneous (over a model in which it can vary from month to month) should be chi-square on 11 degrees of freedom (11 is the change in the number of model parameters) under the null hypothesis of homogeneity. In the surprising event of the test result conflicting with the decision reached in Sec. 4, we recommend the likelihood ratio result as the more reliable, due to its more rigourous justification. In this instance, we would have to be satisfied that the preliminary analysis of Sec. 4 has at least allowed us to get to this stage, while proving to be somewhat misleading!

Notice that once the thresholds and the termination interval have been chosen, a separate seasons model which allows the shape parameter to vary from month to month is in fact equivalent to a model in which each season is treated entirely separately, i.e., no further homogeneities are incorporated. If the extremes occurring in some seasons are not truly large values, then including these seasons in any further analysis will contribute little to return level estimation.

### 5.2 Graphical Evaluation

The overall fit of the separate months model for the magnitudes of excesses over thresholds can be verified via probability plots or quantile plots (plots of fitted distribution function versus empirical distribution function, or fitted quantile versus empirical quantile; the plotting points being defined by the cluster peak exceedances). By using the fitted parameter values to transform each monthly set of CPEs to a common margin (say unit exponential), the fit to all seasons can be assessed simultaneously.

### 5.3 Implementation for the Sheffield Data

Tables 1 and 2 contain thresholds *u_m_* and maximum likelihood estimates for *σ_m_* and *k*(*= k_m_* for all *m* = 1,…, 12) for the separate months model fitted to the CPEs obtained from z* = 30 h and z* = 60 h, respectively.

Likelihood ratio tests confirm the validity of the homogeneous *k* assumption: for the cases z* = 30 h and z* = 60 h, respectively, 8.23 and 7.56 are compared with a chi-square distribution on 11 degrees of freedom; no evidence that *k* should vary from month to month.

**Fig. 3:**
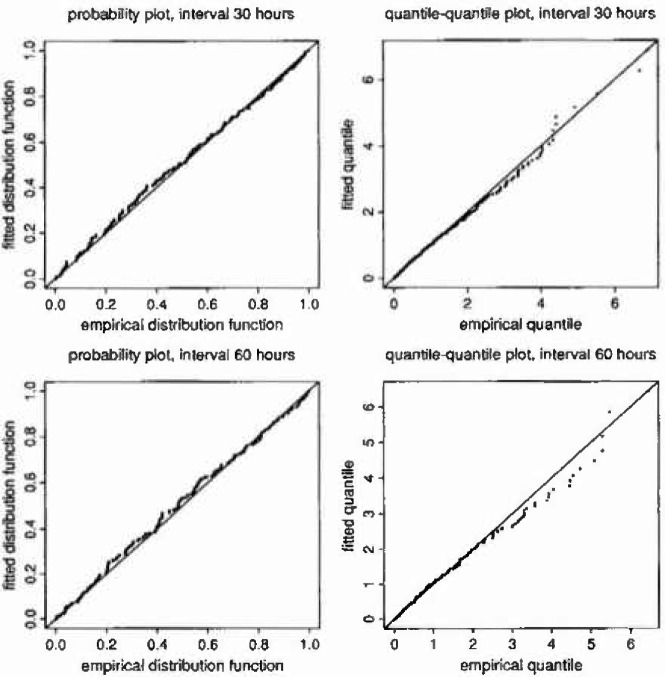
Probability and quantile-quantile plots.

The overall adequacy of the model in both instances is strongly supported by the probability and quantile plots shown in Fig. 3.

## 6. Step 4 — Return Level Estimation

### 6.1 Profile Likelihood Confidence Intervals

For given monthly thresholds *u_m_* and GPD parameters *σ_m_* and *k_m_*, *m* = 1, …, 12, the *r* year return level *q_r_* is obtained as the solution of the equation

$$\sum_{m=1}^{12}\lambda_{m}{\left[1-k_{m}\left(q_{r}-u_{m}\right)/\sigma_{m}\right]}^{1/k_{m}}=r^{-1},$$ (4)

where λ*_m_* is the monthly exceedance rate of threshold *u_m_*. This arises by setting the exceedance rate of level *q_r_* in any given year, given by the LHS in Eq. (4), equal to 1/*r*, (Note that if *q_r_ ≤ u_m_* for any *m*, then the quantity $\lambda_{m}{\left[1-k_{m}\left(q_{r}-u_{m}\right)/\sigma_{m}\right]}^{1/k_{m}}$ should be replaced by *λ_m_*; and if for any *m k_m_* > 0 and *q_r_ ≥ u_m_* + *σ_m_*/*k_m_*, the replacement should be by zero, because of the range on which the GPD is defined.)

We have not yet considered the monthly exceedance rate parameters *λ_m_*. Assuming a Poisson rate of storm occurrence (following Ref. [7]), the maximum likelihood estimates for these are simply the mean annual numbers of storms occurring in each month. A point estimate for *q_r_* can be obtained by substituting the thresholds *u_m_*, and the parameter estimates for *λ_m_*, *σ_m_*, and *k_m_* into Eq. (4), and solving numerically. Standard errors can be estimated via techniques such as the delta-method, but the construction of symmetrical confidence intervals within a specified number of standard errors either side of the mean is not recommended. Instead, we strongly suggest the use of profile-likelihood. Rather than use the limiting quadratic form of the likelihood surface, profile-likelihood makes use of its actual shape for the data in question. The severe asymmetry of the surface often encountered when it is calculated for return levels suggests that conventional symmetrical confidence intervals are highly misleading.

The details involved in the calculation of profile-likelihood confidence intervals for return levels are not entirely straightforward, and we describe them here. For each of a range of possible values of the *r* year return level *q_r_*, we maximize the log-likelihood with respect to the model parameters subject to the constraint Eq. (4), which ensures that *q_r_* is in fact the desired return level. Technically this can be achieved by making one of the parameters the subject of Eq. (4). Suppose, without loss of generality, that *σ*_1_ is chosen. Then Eq. (4) gives

$$\sigma_{1}=k_{m}\left(q_{r}-u_{1}\right)/\left\{1-{\left[r^{-1}-C)/\lambda_{1}\right]}^{k_{m}}\right\},$$ (5)

where

$$C=\sum_{m=2}^{12}\lambda_{m}\left[1-k_{m}\left(q_{r}-u_{m}\right)/\sigma_{m}\right].$$ (6)

The return level *q_r_* is fixed at the desired level, and the log-likelihood *L ≡ L*(*q_r_*) maximized with respect to the parameters λ*_m_*, *k_m_*, and *σ*_2_, …, *σ*_12_. At each iteration in the maximization, *σ*_1_ is calculated numerically from Eq. (5), and *L* is obtained as follows: suppose the CPEs occur over a period of *l* years, and the number of CPEs in month *m* in year *j* is *n_mj_*. Let $n_{m}=\sum_{j=1}^{l}n_{mj}$, and denote the CPEs *y_mi_*; *i* = 1, … *n_m_*. Then

$$\begin{array}{l} L=\sum_{m=1}^{12}\left[-n_{m}\;\log\;\sigma_{m}+\left(\frac{1}{k_{m}}-1\right)\sum_{i=1}^{n_{m}}\log\left(1-\frac{k_{m}y_{mi}}{\sigma_{m}}\right)\right]\\ -l\sum_{m=1}^{12}\lambda_{m}+\sum_{m=1}^{12}n_{m}\;\log\;\lambda_{m}-\sum_{m=1}^{12}\sum_{j=1}^{l}\log\left(n_{mj}1\right). \end{array}$$ (7)

A confidence interval for *q*, can then be formed via inversion of a likelihood ratio test, i.e. as the set of values *q*_0_ for which $2\left[L\left({\hat{q}}_{r}\right)-L\left(q_{0}\right)\right]$ is not significant when compared with a chi-square distribution on one d.f., where ${\hat{q}}_{r}$ is the m.l.e. for *q_r_*.

### 6.2 Implementation for the Sheffield Data

Tables 3 and 4 give point estimates and 95% profile-likelihood confidence intervals for the 10, 50, and 1000 year return levels at the Sheffield site, using the CPE sets obtained via z* = 30 h and z* = 60 h, respectively.

**Fig. 4:**
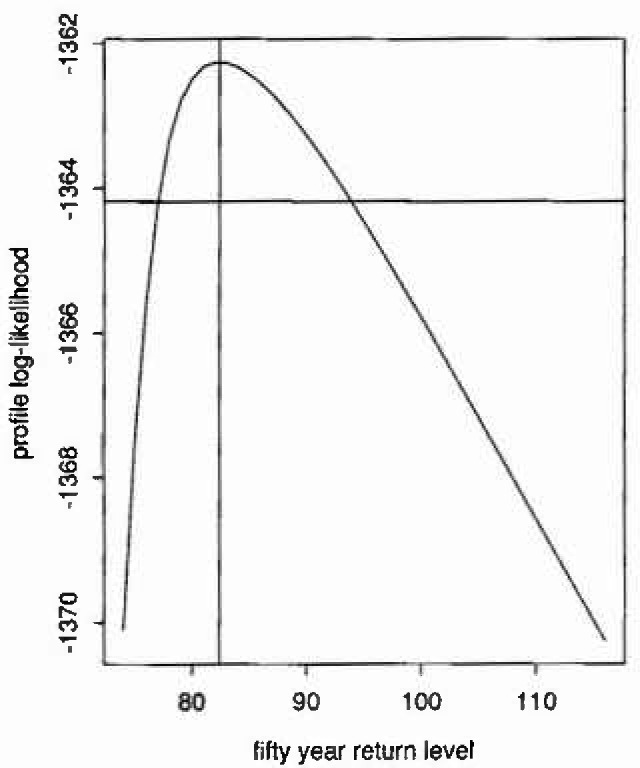
Profile likelihood for 50 years return level.

Figure 4 shows the profile-likelihood for *q*_50_ obtained using z* = 30 h, illustrating the gross asymmetry in the surface. The vertical line is plotted through ${\hat{q}}_{50}=82.4$ knots. The horizontal line lies at a level $0.5\times {\chi_{1}}^{2}\left(0.95\right)$ below the maximized log-likelihood, the intersections with the surface thus providing the bounds for the 95% confidence interval.

## 7. Discussion

The analysis of the previous four sections seems to be very satisfactory for the data collected at Sheffield. Theoretically motivated models appear to be vindicated by the good fit demonstrated by the plots, and homogeneity arguments pertaining to the wind process in different seasons are supported. The consistency of inferences drawn from the two sets of CPEs obtained using z* =30 h and z* = 60 h suggests a robustness of results to the informal methods employed in the selection of thresholds and in cluster identification procedures. Finally, while the entire recommended procedure may appear quite complex, once the appropriate software has been set up it can be implemented very quickly and easily, even on a small machine such as a Sun SPARC station.

Despite the success of the algorithm described, which it is expected will be repeated at other sites, it is *very* important to bear in mind a number of cautionary comments. In particular, we must remember that we have relied very heavily on the assumption that there is essentially a single meteorological mechanism which is responsible for the generation of all extreme gusts. It is clear that this is *violated* in climates where several distinct types of storm can generate extreme winds (e.g., both normal temperate zone storms and hurricanes can occur and generate very high velocity winds). At sites at which such climates prevail, considerations different to those presented in this paper apply. For example, it may be that we know that hurricanes *can* occur at a site, but the short run of data available does not include any hurricanes. This highlights a basic limitation in any extreme value analysis—*if we cannot assume that all the physical mechanisms which can generate extremes have been observed in our data, we cannot produce realistic estimates for return levels*. The best we could do under such circumstances is attempt to import knowledge on the unobserved mechanisms from other sites. Any such analysis would, of course, be extremely vulnerable to inter-site differences in behaviour, which could only be assessed theoretically.

In the more favourable situation where instances of all the relevant types of system have been observed, it seems clear that *separate models* should be fitted to the extremes generated by each one. The overall exceedance rate of any particular high level could then be expressed as a sum of components corresponding to each system type, and return levels estimated numerically in a manner similar to that employed in Sec. 6.

Two further aspects of the models considered here are worth brief discussion:

### 7.1 Piecewise Seasonality

The discontinuous (piecewise) nature of the manner in which all seasonally varying parameters are modelled clearly does not match the *continuous* change inherent in natural processes. However, experimentation with model modifications which allow the parameters to vary continuously [10], suggests that inferences are barely altered in relation to a separate months model for extreme wind gusts. The significant increase in computation time incurred by fitting continuously varying parameters is therefore not thought to be worthwhile.

### 7.2 Weibull-Type Tails

More interestingly, we note that the shape parameter *k* fitted to the Sheffield data is very definitely *positive*. A likelihood ratio test overwhelmingly rejects a null hypothesis which constrains *k* to be zero, in favour of an alternative which allows it to be greater than zero.

Positive *k* values correspond to a Weibull-type upper tail (with a finite upper endpoint) for the distribution of extremes. Traditional analyses, on the other hand, have been based on the assumption of a Gumbel-type upper tail for extreme wind speeds (with no upper endpoint), following from the notion that there is no natural upper bound to wind velocity anywhere near the orders of magnitude at which wind-speeds are actually observed. However, the findings of this paper concur with those of many other authors. Lechner, Leigh, and Simiu [5], for example, find that a Weibull distribution performs significantly better than a Gumbel distribution for the majority of a sample of 100 stations studied in the United States. These authors point out that convergence to the Gumbel distribution can be *extremely* slow, and that the Weibull distribution, as a penultimate asymptotic approximation, can then often provide a better fit even for sample sizes as large as one billion. In view of this consideration, we contend that the arguments supporting the use of the Gumbel distribution are something of a red herring as far as any practical applications are concerned, and that if the data supports the case for Weibull-type upper tails, then a positive shape parameter should duly be fitted!

### 7.3 Conclusions

The analysis of the Sheffield data presented in this paper has stood up to a fairly rigourous scrutiny. Further, the assumption of a single meteorological mechanism underlying the generation of extreme gusts is believed to be well-founded in the U.K., and we suggest that even the estimates of 1000 year return levels produced from the 10 years of data can be quoted with some confidence (provided that we remember that the quotation of a 1000 year return level does not incorporate any forecast of a homogeneous climate over the next 1000 years!). It is worth elaborating here on the precise manner in which the *extreme value paradigm* [1] has been applied to our problem of return level estimation. Theoretical (asymptotic) arguments suggest that the GPD should provide a good *approximation* to cluster peak excesses over thresholds, provided the thresholds are *large enough*. Since the approximation *does* appear to be good for all thresholds above a level close to the upper 93rd percentile of the data, we feel justified in assuming that the asymptotic arguments are applicable at these levels. By their very nature, they are then applicable at all higher levels. This enables us to *extrapolate* beyond the upper endpoint of our sample, and hence estimate return levels for periods far longer than those for which data have been recorded. There is obviously a limit to the extent to which this extrapolation is viable, but hopefully this should be self-apparent: provided the method of calculating confidence intervals is not based on unfounded assumptions about the shape of the likelihood surface, any attempt to extrapolate *too* far will simply lead to confidence intervals which are too wide to be of use.

However, this last point leads to a very important cautionary note. Most of the analyses on which current design-level specifications are based make the assumption of Gumbel-type upper tails. The effect of this has almost certainly been to *over-estimate* return levels at most sites. Thus structures have often been designed to be *stronger* than is actually necessary, and the precision of return level estimates has not been of crucial importance. In converting to the more appropriate Weibull-type tails, it becomes *essential* to make adequate allowance for the margins of error associated with return level estimation. To rely, for example, on a procedure such as the delta-method, which does not capture the inherent asymmetry in these error margins, could prove disastrous!

## Figures and Tables

**Table 1:** Results when the separale months model is fitted to cluster peak exceedanccs obtained using z* = 30 h

Month (*m*)	*u_m_*	${\hat{\sigma }}_{m}$	*k*
1	38.38	16.75 (2.01)	
2	29.68	15.60 (1.99)	
3	34.65	11.37(1.39)	
4	2937	10.63 (1.25)	
5	24.85	7.68 (0.79)	
6	25.77	8.75 (0.96)	0.3603 (0.0469)
7	24.26	7.23 (0.79)	
8	23.71	9.22 (1.08)	
9	29.95	12.12 (1.37)	
10	2932	10.76 (1.26)	
11	34.45	12.34 (1.53)	
12	33.27	16.03 (1.84)	

**Table 2:** Results when the separate months model is fitted to cluster peak exceedanccs obtained using z* = 60 h

Month (*m*)	*u_m_*	${\hat{\sigma }}_{m}$	*k*
1	39.95	23.28 (2.60)	
2	30.94	22.93 (2.60)	
3	35.77	14.09 (1.57)	
4	30.52	13.61 (1.5t)	
5	25.65	9.48 (0.95)	
6	26.52	11.52 (t.20)	0.4975 (0.0573)
7	25.07	8.76 (0.90)	
8	24.45	11.97 (1.30)	
9	31.03	16.75 (1.89)	
10	30.69	13.57 (1.44)	
11	35.58	16.32 (1.98)	
12	34.75	19.98 (2.1t)	

**Table 3:** Point estimates and 95% profile-likelihood confidence intervals for some return levels: z* =30 h

Return period and m.l.e. for return level		
$10\;\left({\hat{q}}_{10}=76.4\right)$	$50\;\left({\hat{q}}_{50}=82.4\right)$	$1000\;\left({\hat{q}}_{1000}=88.8\right)$

95% Profile-likelihood confidence interval		

(72.0, 84.9)	(77.0, 93.9)	(81.8, 103.8)

**Table 4:** Point estimates and 95% profile-likelihood confidence intervals for some return levels: z* = 60 h

Return period and m.l.e. for return level		
$10\;\left({\hat{q}}_{10}=77.3\right)$	$50\;\left({\hat{q}}_{50}=82.5\right)$	$1000\;\left({\hat{q}}_{1000}=82.8\right)$

95% Profile-likelihood confidence interval		

(73.3, 86.7)	(78.4, 93.1)	(82.3, 97.8)
